# Spherical Cellulose Micro and Nanoparticles: A Review of Recent Developments and Applications

**DOI:** 10.3390/nano11102744

**Published:** 2021-10-17

**Authors:** João P. F. Carvalho, Ana C. Q. Silva, Armando J. D. Silvestre, Carmen S. R. Freire, Carla Vilela

**Affiliations:** Department of Chemistry, CICECO—Aveiro Institute of Materials, University of Aveiro, 3810-193 Aveiro, Portugal; joao.pedro.carvalho@ua.pt (J.P.F.C.); ana.cristina.silva@ua.pt (A.C.Q.S.); armsil@ua.pt (A.J.D.S.); cfreire@ua.pt (C.S.R.F.)

**Keywords:** cellulose, cellulose derivatives, sphere-shaped particles, microparticles, nanoparticles, particle manufacturing, emulsification, microfluidics, nanoprecipitation

## Abstract

Cellulose, the most abundant natural polymer, is a versatile polysaccharide that is being exploited to manufacture innovative blends, composites, and hybrid materials in the form of membranes, films, coatings, hydrogels, and foams, as well as particles at the micro and nano scales. The application fields of cellulose micro and nanoparticles run the gamut from medicine, biology, and environment to electronics and energy. In fact, the number of studies dealing with sphere-shaped micro and nanoparticles based exclusively on cellulose (or its derivatives) or cellulose in combination with other molecules and macromolecules has been steadily increasing in the last five years. Hence, there is a clear need for an up-to-date narrative that gathers the latest advances on this research topic. So, the aim of this review is to portray some of the most recent and relevant developments on the use of cellulose to produce spherical micro- and nano-sized particles. An attempt was made to illustrate the present state of affairs in terms of the go-to strategies (e.g., emulsification processes, nanoprecipitation, microfluidics, and other assembly approaches) for the generation of sphere-shaped particles of cellulose and derivatives thereof. A concise description of the application fields of these cellulose-based spherical micro and nanoparticles is also presented.

## 1. Introduction

The inception of micro and nanotechnology has brought society to a new era, opening the gates to a whole “new world” of tremendous potential. By manipulating materials at these scales, scientists are now able to precisely manufacture very small particles with customizable attributes, meticulously optimized for specific applications [1,2]. These applications include, for example, the delivery of pharmaceuticals [3], agrochemicals [4], and bioactive ingredients and nutraceuticals [5], as well as cell culture [6], cancer treatment [7] and enzyme immobilization [8], but also environmental remediation [9,10], electronics [11], and energy conversion and storage [12,13].

Most of the micro and nanoparticles have a composition based on inorganic and organic compounds, synthetic polymers, or hybrid materials [2,14,15,16,17,18,19,20]. Nevertheless, and just like in other fields of modern science and technology, there is a growing interest in using alternative bio-based raw-materials for the production of micro and nanoparticles, because renewable resources are essential to contribute for the goals of the 2030 Agenda for Sustainable Development [21]. So, natural polymers, produced by living organisms, are particularly relevant to engineer micro and nanostructures, as reviewed by Joye & McClements [1] and, more recently, by Stanisz et al. [22]. In fact, particles have already been obtained from polysaccharides (or derivatives thereof) [19], namely cellulose [23], alginate [24] and chitosan [25,26], but also from proteins [18], such as albumin [27], gelatin [28] and fibroin [29], and nucleic acids (e.g., DNA [30] and RNA [31]).

Among the existing natural polymers, the ubiquitous and inexpensive cellulose with a cost of ca. 790 € per metric ton or 0.79 € kg^−1^ (wood pulp) [32], is exceptionally interesting for particle development due to its renewability, biocompatibility, good mechanical performance, and customizable surface chemistry. In practice, there are already commercial products of cellulose-based micro and nanoparticles. For instance, the JNC Corporation is commercializing the Cellufine™, viz. cellulose spherical beads with particle size of ca. 40–130 µm, which are used as chromatography media designed for the purification of proteins, enzymes, and other biomolecules [33]. The company IONTOSORB^®^ produces Macroporous Bead Cellulose MT, i.e., highly porous regenerated cellulose with particle size of ca. 30–250 µm, for application as gel filtration media for biomolecule separations [34]. The Cytiva™ technologies is selling the Cytopore™ macroporous microcarriers—crosslinked cotton cellulose particles with diameters of 200 to 280 μm—that are designed for use in stirred suspension culture systems for the growth of cells and the production of recombinant proteins for therapeutic use, as well as for the immobilization of insect cells, yeast, and bacteria [35]. Also worth mentioning is the fact that, in addition to spherical particles, cellulose elongated nanostructures, viz. cellulose nanocrystals with cross-sectional size of 5–70 nm and length in the range 100–250 nm [36], are being commercialized by the Canadian CelluForce under the tradename CelluForce NCC^®^, for application as interface stabilizers, rheological modifiers, films/coatings, reinforcing additives, among other examples [37].

The production of small sphere-shaped particles requires specific, laborious, and complex fabrication techniques, some of which were recently discussed by Zielińska et al. [38] who reviewed the most commonly used methods for the production of nanoparticles from synthetic polymers, whereas Joye & McClements [1] examined the top-down and bottom-up fabrication methods for biopolymer-based nanoparticles and microparticles. In the specific case of the cellulose natural polymer, these methodologies are mostly based on the dissolution, regeneration and shaping of cellulose or its derivatives [23,39]. Understandably, the selection of an appropriate technique will be heavily dependent on the starting material, the desired particle size and surface characteristics, and the final application.

A substantial number of publications about the assembly of sphere-shaped particles from cellulose and its derivatives has surfaced. However, and as far as our literature analysis could discover, the last literature reviews in this matter date back to 2013 and 2015. The first is the comprehensive appraisal by Gericke, Trygg & Fardim [39] dealing with the preparation, characterization, and applications of functional cellulose beads (spherical particles exclusively composed of cellulose) with diameters in the micro to millimeter scale (≥10 μm). The second is the review by Zhao and Winter [23] dedicated to the available methods for nanosphere formation based on cellulose and its derivatives. Although these publications are of great interest for scientists working in chemistry, biochemistry, materials science, and other related areas, a concise and up-to-date portrayal is essential to map a path of the latest works on microparticles and nanoparticles from cellulose.

In this manner, the present review comprises some of the most recent and relevant works on the manufacturing of sphere-shaped cellulose-based micro and nanoparticles. Herein, and for clarity purposes, micro and nanoparticles solely or mostly composed of cellulose (or its derivatives) are microspheres/microcapsules with diameters at the microscale, and nanospheres/nanocapsules with diameters at the nanoscale, respectively. Therefore, cellulose nanocrystals with a rod-like morphology (elongated nanostructures), cross-sectional size of 5–70 nm and length in the range 100–250 nm [36,40,41,42,43], despite being considered cellulose nanoparticles [44], are out of the scope of this appraisal. In terms of organization, the current review includes a concise overview of the cellulose fundamentals and the fabrication methodologies of spherical particles, followed by the landscape of available examples dealing with the production of sphere-shaped micro and nanoparticles derived from the ubiquitous cellulose. The main current applications of these particles are also briefly covered.

## 2. Cellulose Fundamentals

**Figure 1:**
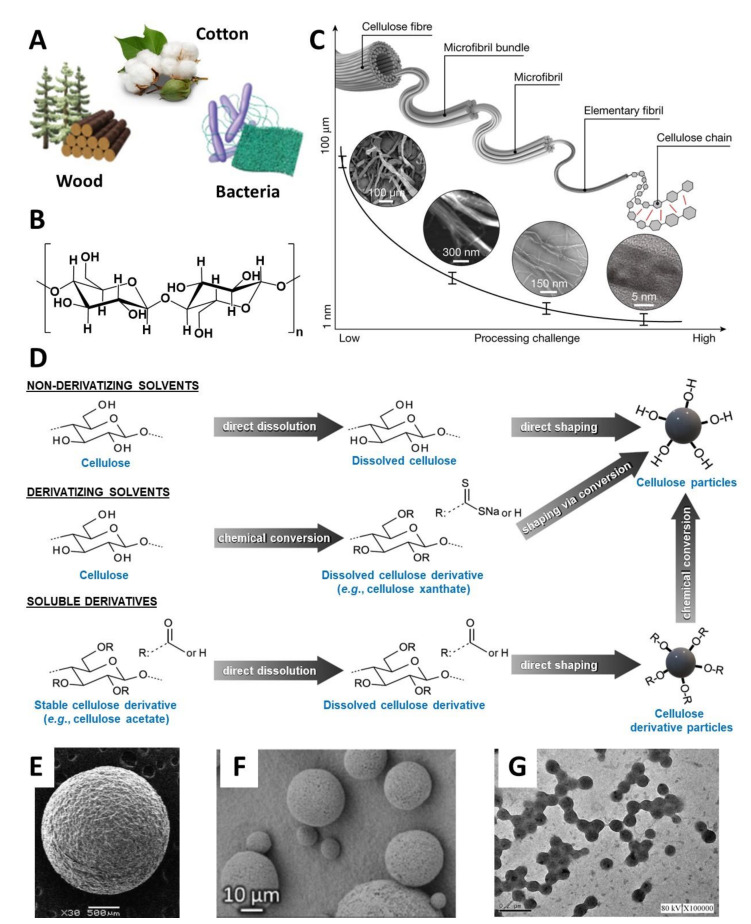
(**A**) Common sources of cellulose (Adapted with permission from [76]. Copyright Springer Nature, 2021), (**B**) chemical structure of cellulose, (**C**) schematic description of the hierarchical structure and manufacturing challenge of cellulose (Reproduced with permission from [76]. Copyright Springer Nature, 2021), (**D**) general pathways for cellulose dissolution and particle shaping (Adapted with permission from [39]. Copyright American Chemical Society, 2013), and electronic micrographs of (**E**) a cellulose bead (Reproduced with permission from [92]. Copyright Elsevier, 2012), (**F**) cellulose microparticles (Reproduced with permission from [93]. Copyright American Chemical Society, 2020), and (**G**) cellulose nanoparticles (scale bar: 200 nm, Reproduced with permission from [94]. Copyright Elsevier, 2018).

Cellulose is the most abundant natural polymer in the planet, and it is characteristically present in woody substrates and plant-life (Figure 1A), where it is associated with other cell-wall constituents (like hemicellulose and lignin) and plays a key-role in plant support [45]. This natural polymer is also produced by tunicates, algae, and non-pathogenic bacteria [36]. Regardless of the source, cellulose is comprised of D-glucopyranose units linked by β(1→4) glycosidic bonds (Figure 1B). The characteristic intra- and intermolecular hydrogen bond network of this polysaccharide is responsible for the crystallinity of cellulose, its insolubility in water and in most common organic solvents, and the three-dimensional arrangement of cellulose into microfibrils (with diameters of 2–20 nm, Figure 1C). These microfibrils, comprised of both crystalline and amorphous domains, further entangle to form macrofibrils [46,47].

Although cellulose is mainly used in the pulp and paper industry, it has been exploited, along with its derivatives (e.g., cellulose acetate (CA), carboxymethylcellulose (CMC), and ethylcellulose (EC)) and nanoforms (i.e., cellulose nanocrystals (CNCs), cellulose nanofibrils (CNFs) and bacterial nanocellulose (BNC) [36,48,49]), for other purposes, including textiles [50], fuel cells [51,52,53], electronics [54,55], water remediation [56,57,58], food packaging [59,60,61], cosmetics [62,63], drug delivery [64,65], cell and tissue cultures [66,67], just to mention some examples. In order to be applied in these multiple domains, cellulose needs to be extracted from biomass resources by conventional technologies (e.g., kraft pulping) or innovative methodologies (e.g., extraction with ionic liquids (ILs) or deep eutectic solvents (DES)) [68,69,70,71] or produced in its pure form by non-pathogenic bacteria (e.g., *Komagataeibacter*) [63,72,73]. Then, it can be processed into blends, composites, and hybrid materials in the form of membranes, films, coatings, hydrogels, foams, and particles [74,75,76,77,78].

One of the main difficulties when processing cellulose is associated with its insolubility in most common solvents [47]. The approaches developed to dissolve cellulose are usually classified as either derivatizing or non-derivatizing solvents. The derivatizing options act through the modification of cellulose [79,80], while non-derivatizing solvents act by dissolving the polysaccharide directly (Figure 1D). Examples of these solvents include the use of aqueous solutions of transition metals (e.g., cuprammonium hydroxide), or bases (e.g., NaOH or LiOH) [81,82,83]. Amongst non-aqueous alternatives, the use of lithium chloride (LiCl) and *N*,*N*-dimethylacetamide (DMA) systems [83,84], and the dissolution of cellulose with *N*-methylmorpholine-*N*-oxide (NMMO) are described [68,79]. An overview of traditional solvents for cellulose dissolution can be found in the relevant literature [39,79,85,86]. Major advances in this domain that can be explored to overcome these constraints include the utilization of switchable solvents (e.g., 1,8-diazabicyclo[5.4.0]undec-7-ene (DBU)/CO_2_) [87] or organic electrolyte solutions (i.e., mixtures of a room-temperature ionic liquid with a neutral, organic, polar co-solvent (e.g., dimethyl sulfoxide (DMSO)) [88,89], for cellulose green and safe dissolution (and chemical conversion). Furthermore, the dilemma of cellulose dissolution can also be circumvented by chemical modification into its derivatives (Figure 1D), such as cellulose esters (e.g., CA), or ethers (e.g., CMC, methyl cellulose (MC), EC and hydroxyethyl cellulose (HEC)), which are generally soluble in water and in common organic solvents [90].

Cellulose can be used as a substrate to manufacture sphere-shaped beads, microparticles, and nanoparticles with diameters at the millimetric, micrometric, and nanometric scales, respectively [23,39,91]. As an illustrative example, Kim et al. [92] developed cellulose hydrogel beads with a diameter of ca. 2.0 mm for the immobilization of lipase from *Candida rugosa*, as shown in Figure 1E. In more recent studies, Druel et al. [93] produced cellulose aerogel microparticles with diameters in the range of 5.4 ± 1.8 μm to 20.9 ± 8.9 μm via emulsion-coagulation technique (Figure 1F), while Chin et al. [94] fabricated cellulose nanoparticles with diameters ranging between 70 and 365 nm by the nanoprecipitation method (Figure 1G).

In the present review, focus will be placed only on sphere-shaped cellulose-based micro and nanoparticles. The fabrication of these particles requires specific and complex fabrication techniques, since the processing challenge increases with size reduction, as illustrated in Figure 1C. Therefore, selecting an appropriate manufacturing technique to produce sphere-shaped cellulose microparticles and nanoparticles is an intricate work of balance between the specificities of cellulose or derivatives thereof, the desired particle features, and the intended application, as discussed in the following sections.

## 3. Overview of Spherical Particles Fabrication

The methods for the production of spherical particles depend greatly on the type of starting raw material, namely inorganic and organic compounds, or synthetic and natural polymers [1,95,96,97]. In the particular case of polymeric micro and nanoparticles, some of the go-to strategies include the following: (i) emulsification, nanoprecipitation, dialysis, and supercritical fluid technology, as reviewed by Crucho & Barros [98] and Zielińska et al. [38] for synthetic polymers, and (ii) shredding, homogenization, extrusion, anti-solvent precipitation, coacervation, inclusion complexation, and fluid gel formation, as revised by Joye and McClements [1] for biopolymer-based nanoparticles and microparticles.

From the perspective of this review, the following paragraphs provide a concise exposition of the production methodologies used to fabricate spherical cellulose-based particles, namely emulsification, nanoprecipitation, microfluidics, and other assembly approaches. These methodologies can generate both spheres, viz. particles with a polymer matrix-like structure where the polymer and other components are uniformly dispersed, and capsules, i.e., particles with a core-shell morphology in which the polymer shell surrounds the confined components in the inner cavity (aqueous or oily) [98].

**Figure 2:**
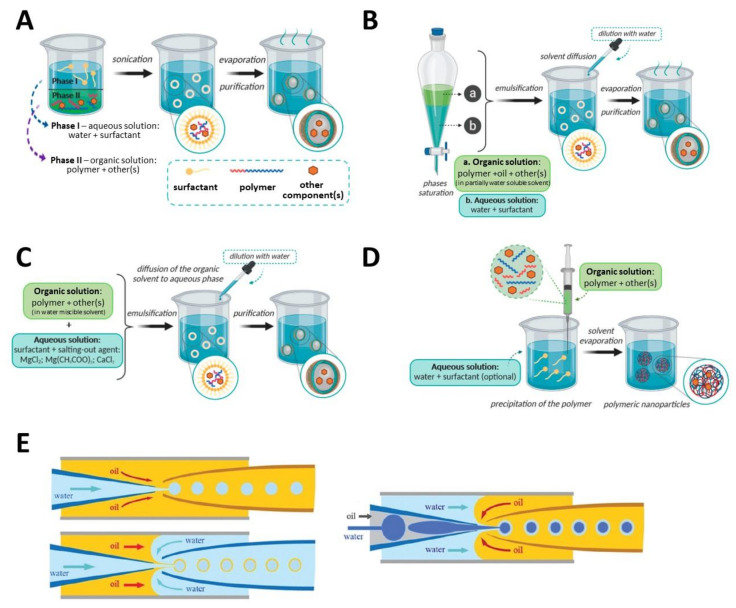
Schematic representation of different methods for spherical particle production: (**A**) emulsification/solvent evaporation, (**B**) emulsification/solvent diffusion, (**C**) emulsification/reverse salting-out, (**D**) nanoprecipitation (Adapted with permission from [38]. Copyright MDPI, 2020), and (**E**) microfluidics devices for emulsion droplets (Reproduced with permission from [105]. Copyright Royal Society of Chemistry, 2018).

Emulsions consist in the mixing of two (or more) liquid phases, which are totally or partially immiscible in one another, with the aid of surfactants, i.e., amphiphilic surface-active molecules that stabilize the interfacial tension between the two liquids [98]. Typically, emulsion systems are formed by the dispersion of an oil phase in an aqueous phase (oil-in-water, *o*/*w*) or vice-versa (water-in-oil, *w*/*o*), or even more complex systems such as water-in-oil-in-water (*w*/*o*/*w*). The particles are then obtained as aqueous colloidal suspensions through the (i) evaporation of the solvent (emulsion/solvent evaporation technique, Figure 2A), (ii) dilution with a large volume of water, inducing solvent diffusion (emulsion/solvent diffusion technique, Figure 2B), or (iii) solvent diffusion through the salting-out effect (emulsification/reverse salting-out technique, Figure 2C) [38]. These techniques usually originate nanoemulsions (10–100 nm), miniemulsions (100–1000 nm) and macroemulsions (>1 μm) depending on the droplet size [99], and thus are suitable to produce particles (spheres and capsules) at the micro and nano scale ranges. Although these techniques are relatively simple, economical and allow the easy control of particle size and size distribution, they are disadvantaged from high energy consumption, the use of surfactants and organic solvents, and long purification processes [23]. Supplementary facts and details about emulsification processes are available elsewhere [38,98,99].

The nanoprecipitation, also known as solvent displacement technique, is a methodology commonly used to fabricate nanoparticles, as an alternative to the emulsion process. Here, the polymer is first dissolved in an adequate solvent, and posteriorly added in a one-step or drop-wise fashion to an antisolvent (miscible with the polymer solvent), as illustrated in Figure 2D [98]. The solvent subsequently diffuses into the antisolvent, causing the precipitation of the polymer, in the form of nanoparticles with well-defined size [100]. In terms of advantages, the nanoprecipitation is a one-step and economical technique that presents excellent reproducibility, does not require the use of surfactant and allows the efficient entrapment of target molecules; still, the low concentration of particles achievable is the main drawback [23]. Additional information about the nanoprecipitation technique is reported in the appropriate literature [38,98,100,101,102].

The microfluidics technology is a versatile chip-platform that enables the design of microparticles with adjustable size, shape, and morphology through the precise manipulation of multiphasic flows at the microscale [103]. In fact, microfluidics has been used to assemble a diversity of designs, such as spherical, tubular, and helical with Janus or core-shell structures, by regulating microchannels geometry, precursor solutions, and hydrodynamic fluids flow rates [104]. In the droplet microfluidics (one of the most effective techniques), immiscible liquids (a dispersed phase (droplet phase) and a continuous phase) are conducted through distinct microchannels (Figure 2E) [105]. In terms of advantages, microfluidics offers size and process control, small particle size, and monodispersity [23]. Comprehensive discussions about microfluidic technology are accessible in the relevant literature [105,106,107,108], including the review by Liu et al. [109] that discusses emulsification via microfluidic processes and the appraisal by Jo & Lee [103] about biopolymer microparticles fabricated by microfluidics for biomedical applications.

A body of recent research suggests that, apart from emulsification, nanoprecipitation, and microfluidics, sphere-shaped polymeric particles can also be prepared by other methodologies, such as layer-by-layer (LbL) assembly [110], supercritical fluid technology [98], spray-assisted techniques, such as electrohydrodynamic atomization [111], or even a combination of some of the previously enumerated approaches to engineer more complex shapes and morphologies.

## 4. Production of Spherical Cellulose-Based Microparticles

The production of sphere-shaped cellulose-based microparticles can be performed by emulsification processes [93,112,113,114,115], and microfluidics technology [116,117,118,119,120,121,122,123,124,125], as well as other less common techniques, namely spray-assisted techniques [126,127,128,129,130], and the LbL assembly [131,132]. Concerning the cellulosic substrate, the majority of the studies reported the utilization of cellulose derivatives, such as CMC [120,131], CA [114,119] and EC [123,124,133,134], but also pristine vegetable cellulose [112,135,136,137], bacterial nanocellulose [116,117,118] and microcrystalline cellulose (MCC) [93,115,138,139]. The preference for cellulose derivatives to generate microparticles was anticipated given their solubility in water or in most common organic solvents, which translates into simpler processability.

Some of the most recent and important contributions about microparticles composed of cellulose and derivatives thereof via different production methods are discussed in the subsequent sections. Depending on the method, both microspheres (i.e., particles with a cellulose matrix-like structure) [93,138,140] or microcapsules (i.e., particles with a cellulose core-shell morphology) [116,132,139,141] can be obtained.

### 4.1. Emulsification Processes

Emulsion techniques remain the golden standard in the preparation of sphere-shaped cellulose-based microparticle, as evidenced by the number of publications gathered in Table 1. Cellulose derivatives, such as CA, cellulose acetate butyrate (CAB), cellulose acetate phthalate (CAP), CMC or EC, are largely employed in these techniques, given their simpler solubility. Nevertheless, emulsions have also been applied to prepare microparticles from BNC [113] and CNFs [142], or even from native vegetable cellulose dissolved in NaOH/urea aqueous solutions [112] or microcrystalline cellulose dissolved in ionic liquids [143] (Table 1).

Certain characteristics of the microparticles obtained by emulsification may be controlled with the manipulation of the experimental conditions. For example, the stirring speed seems to play a key role in the size of the particles, as reported by Zhang et al. [142], who observed that an increase from 500 rpm to 1000 rpm caused a 5-fold decrease in the size of a CNFs-based microsphere from ca. 500 µm to the sub-micrometre range. Similarly, in a study conducted by Abbaspoor et al. [141], spherical oil-filled EC capsules with average sizes ranging from the nanoscale (33 nm) to the microscale (400 µm) were produced by varying the stirring speed from 30,000 to 1000 rpm, respectively.

**Figure 3:**
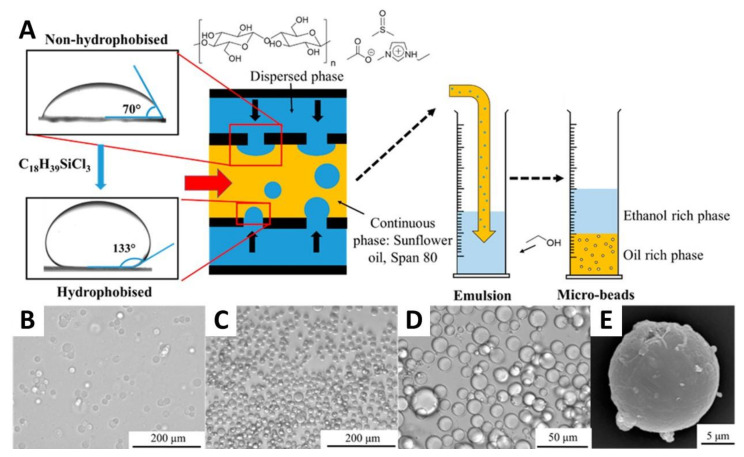
(**A**) Schematic illustration of the membrane emulsification apparatus alongside the contact angles of the disperse phase before and after hydrophobization of the tubular Shirasu porous glass membranes; optical micrographs of the (**B**) emulsion droplets of microcrystalline cellulose, and (**C**,**D**) cellulose microparticles formed via phase inversion with ethanol; and (**E**) SEM micrograph of a cellulose microparticle. Reproduced with permission from [138]. Copyright American Chemical Society, 2017.

In an interesting work, OBrien et al. [138] produced cellulose microparticles by membrane emulsification, which is an efficient, low energy, and scalable emulsion approach, where an emulsion is generated via the permeation of a liquid (i.e., the disperse phase) through the pores of a membrane into another liquid (i.e., the continuous phase) that is flowing perpendicularly to the membrane. A microcrystalline cellulose solution in [C2mim][Ac]:DMSO was driven through a tubular Shirasu porous glass membrane (10 µm pore), forming a stable emulsion in the continuous phase that was later coagulated in ethanol, as depicted in Figure 3A. Here, an increase of the continuous phase flow rate from 1.4 to 2.4 L min^−1^ caused a significant reduction in microparticle size from 65 to 17 μm (Figure 3B–D). The resulting microspheres were then crosslinked with glyoxal, to reduce shrinkage of the particles upon drying and to increase their mechanical strength. Interestingly, this crosslinking also changed their surface from a smooth to a pitted aspect (Figure 3E) [138].

In a different study by Božič et al. [154], the variation of surfactant type affected the surface morphology of EC particles. Here, surfactants such as poly(vinyl alcohol) (PVA) and CMC yielded particles with smooth surfaces, while the use of high- or low-molecular weight methyl cellulose promoted the formation of a wrinkled surface [154]. Furthermore, the morphology of the surface may be also controlled by the solvent choice. For example, EC microparticles obtained from *w*/*o*/*w* emulsions showed a rough surface when EC was dissolved in dichloromethane, while micropores were observed with chloroform dissolution [150]. Additionally, Murakami et al. [140] have found a correlation between the rate of solvent removal and the porosity of EC microspheres. A slow introduction of water for solvent extraction was linked with a decrease in surface area, while the fast addition of water resulted in porous microspheres with an increased surface area of 40.7 ± 2.1 m^2^ g^−1^.

Other features like encapsulation efficiency and the release profile of the particles might be manipulated from the get-go by choosing different starting materials. In a study conducted by Simões et al. [114], particles from different cellulose derivatives, namely EC, CA, CAB and CAP, were obtained by emulsion and tested for eugenol encapsulation, with CA-based microparticles showing a higher encapsulation efficiency and a slower release of this bioactive compound. According to the authors, the differences in the encapsulation efficiencies are credited to the interaction/affinity of eugenol with the cellulose derivatives.

### 4.2. Microfluidics

The microfluidic technology is another methodology that is being used to fabricate sphere-shaped cellulose-based microparticles. Table 2 summarizes some of the most recent examples of micro-sized particles based on pure cellulose [116,117,118,125], and cellulose derivatives, such as CA [119], CMC [120], EC [123,124] and TEMPO-oxidized CNFs [155], produced by microfluidics.

A creative example of the use of microfluidics to produce cellulose microparticles is based on the encapsulation of cellulose producing bacteria inside a core-shell structured microparticle for long-term static culture [116,117,118], which averts the need of using a chemical process to dissolve cellulose. For instance, Yu et al. [116] utilized microfluidics to generate a sacrificial template based on a core-shell structured microparticle formed by an alginate core and agarose shell, for the encapsulation of the *Gluconacetobacter xylinus* bacterium. After incubation of the bacteria-loaded spheres and production of BNC, the hydrogel template was dissolved (with 1% NaOH at 100 °C), resulting in hollow BNC microcapsules with a diameter of ca. 50 µm [116]. In a similar study, Higashi et al. [117] used microfluidics to obtain nanofibrous microspheres composed of BNC biosynthesized by the *Komagataeibacter xylinus* bacterium, which was encapsulated inside microspheres of gelatin. After the removal of the gelatin sacrificial template, BNC microspheres with ca. 250–1000 µm were obtained. Understandably, the variation of the needle gauge from 58 to 32 µm lead to a two-fold reduction of size, and the same decrease was observed by varying the flow rate of the continuous phase from 50 to 1000 µL min^−1^. The authors also compared the BNC microspheres generated by microfluidics with those produced via emulsification method, and the results clearly show the inferiority of the latter method, which originated a larger distribution in particle size [117]. Very recently, Pepicelli et al. [125] took advantage of the same production process to prepare self-grown BNC microcapsules (by *G. xylinus*) with customizable size and monodispersity, which were influenced by the bacteria concentration (1−5 *v*/*v* %), droplet size (150–400 µm), and surfactant type (Span^®^ 85, phosphatidylcholine or β-lactoglobulin).

**Figure 4:**
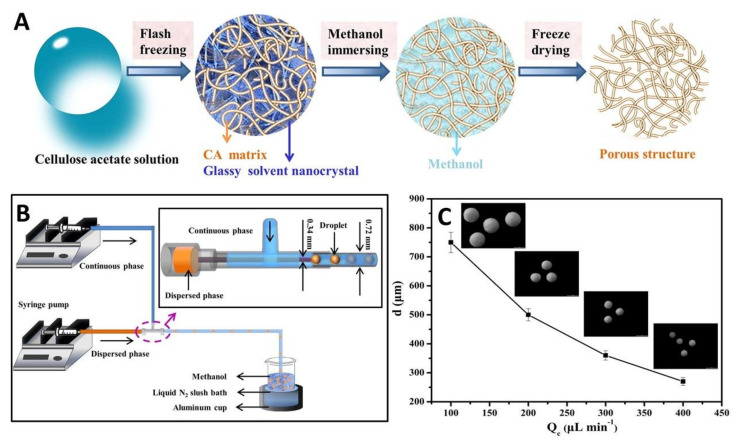
(**A**) Schematic representation of the several steps for the preparation of the porous cellulose acetate (CA) microspheres, (**B**) scheme of the T-junction microfluidic apparatus to fabricate the microspheres, and (**C**) linear fitting of the diameter of the microspheres with different continuous phase flow rate (*Q*_C_) at the fixed dispersed phase flow rate (*Q*_d_) of 10.0 μL min^−1^. Reproduced with permission from [119]. Copyright Elsevier, 2020.

Cellulose microparticles may also be produced via microfluidics from cellulose derivatives that are promptly dissolved in adequate solvents. As an illustrative example, cellulose acetate microspheres with adjustable porosity and size were prepared by Zhang et al. [119] (Figure 4A) using the microfluidics apparatus illustrated in Figure 4B. Their size was found to be dependent on the continuous phase flow rate, as already described above, since an increase from 100 µL min^−1^ to 400 µL min^−1^ resulted in a 3-fold reduction of particle size (Figure 4C). Solvent and polymer concentrations affected the surface area and porosity of the microparticle, with a decrease in porosity linked to higher CA concentration [119].

Interestingly, the microfluidics technology, apart from being used to manufacture micro-sized particles [119] and capsules [156], can also be employed to produce cellulose microfibers (length: 30–100 μm, diameter: 7–45 μm) based on the regeneration of cellulose from ionic liquids [157].

### 4.3. Other Methodologies

Sphere-shaped cellulose-based microparticles can also be prepared by other methodologies, such as the layer-by-layer assembly [131,132] and spray-assisted techniques [126,127,128,129,130]. Examples of cellulose-based microparticles prepared via other methodologies are evidenced in Table 3.

Wang et al. [131] developed microcapsules of CMC and chitosan with a size of ca. 2.15 µm and a shell thickness of 25 nm per bilayer, that retained the spherical shape even after the template removal. The authors stated that the electrostatic interaction between the carboxylic groups of CMC and the ammonium groups of chitosan were the driving force in the LbL assembly. This process was evaluated by UV-Vis spectroscopy using fluorescein isothiocyanate (FITC) labelled chitosan, with an increase in absorbance with the increasing number of bilayers.

In an approach inspired by the plant primary cell-wall composition, Paulraj et al. [132] developed biomimetic microcapsules using layers of modified CNFs, pectin, and xyloglucan via LbL assembly. The use of pectin was crucial for the integrity of the capsule and the retention of a spherical shape after the template removal, and the resulting capsules, with a size of 16 ± 4 µm and a shell thickness of ca. 20 nm, revealed a stimuli-responsive permeability governed by the salt concentration of the medium [132].

In the field of spray-assisted approaches, both electrospray and spray-drying techniques have been used to prepare cellulose microparticles, either with the use of cellulose derivatives or nanocelluloses. The size of the particles obtained in spray-assisted techniques may be manipulated through the adjustment of the experimental parameters of this process (e.g., variation of the flow rate), as well as the evaporation process, namely inlet temperature (temperature of the heated drying gas) [158]. As an example, Guarino et al. [126] studied the electrohydrodynamic atomization of a CA solution containing ketoprofen lysinate by varying the voltage (12–18 kV) and the flow rate of the CA solution (0.5–1.5 mL h^−1^). As expected, the size of the microcapsule drug carriers was influenced by these parameters, with an upsurge in capsule diameter from 287 ± 76 µm to 1248 ± 120 µm as the flow rate increased. On the other hand, the increase of the applied voltage led to a decrease in microcapsules dimensions, but only to a certain extent since voltages higher than 18 kV resulted in size heterogeneities, attributed to some instability of the atomization process [126].

## 5. Production of Spherical Cellulose-Based Nanoparticles

In the domain of sphere-shaped cellulose-based nanoparticles, nanoprecipitation [94,159,160,161,162,163,164,165,166], and emulsification processes [167,168,169,170,171,172,173] are the most used production methodologies, although other less common approaches, namely mechanical treatments [174,175,176,177], chemical and/or enzymatic treatments [178,179,180,181,182], and layer-by-layer assembly [183], can also be used to assemble nanospheres [179,181,182] and nanocapsules [159,168,169,183]. Regarding the cellulosic substrate, most of the studies, as in the case of cellulose-based microparticles, report the use of cellulose derivatives, such as CA [184], CMC [159,168,183] and EC [141,161,169,170,171,172,173,185], although some examples of native cellulose [94,174,175,178], regenerated cellulose [181,182] and MCC [163,164,165] are also available, as discussed in detail in the following paragraphs.

### 5.1. Nanoprecipitation

Nanoprecipitation (or solvent displacement technique) is one the most used techniques to fabricate sphere-shaped cellulose-based nanoparticles, as outlined in Table 4. The direct dissolution of cellulose (from cotton or paper waste) is often made possible with aqueous solutions of NaOH/urea/thiourea, followed by the regeneration into spherical shapes achieved through the drop-wise addition of this solution in an antisolvent (e.g., water or ethanol) [94,162]. For example, Chin et al. [94] tested the impact of cellulose solution concentration and volume ratio of solvent (aqueous solution of NaOH/urea/thiourea)/antisolvent (ethanol) in particle formation via nanoprecipitation. By varying the cellulose concentration from 0.001% to 0.005–0.1% (*w*/*v*), the particle size increased from 218 nm to 263–683 nm. However, the variation in solvent/antisolvent volume ratio from 1:20 to 1:60 decreased the sizes from ca. 365 nm to 70 nm. Hence, such parameters (viz. cellulose concentration or solvent/antisolvent ratio) may be used to tune the size of the resulting particles [94].

The nanoprecipitation method has also been applied using pure cellulose dissolved in alternative solvents, such as ILs [165]. Regarding the parameters that influence this process, the H-bond acidity of the antisolvent has been shown to affect the regeneration of pure cellulose from ILs. When comparing different antisolvents, namely water, methanol, ethanol, and n-propanol, Fan et al. [186] reported the favoured regeneration of this polysaccharide (dissolved in [Bmim]Cl) in water, since water possessed the highest H-bond acidity. The choice of the antisolvent also influenced the properties of the regenerated cellulose nanoparticles, namely the crystallinity, enthalpy in cellulose degradation, and thermal stability [186].

**Figure 5:**
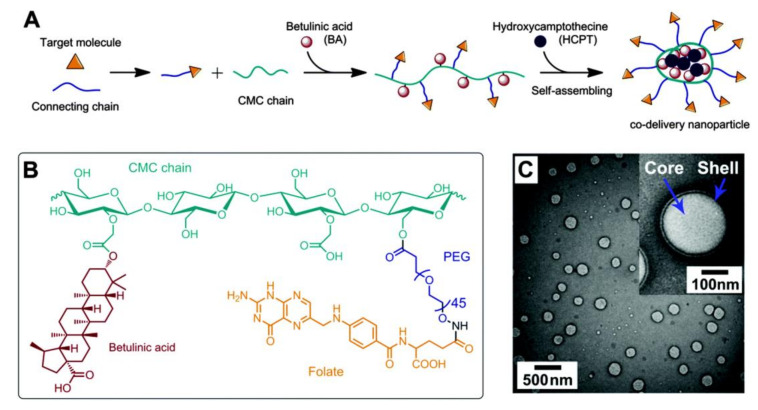
Schematic illustration of (**A**) the synthesis of the folic acid-poly(ethylene glycol)-carboxymethylcellulose-betulinic acid copolymer, followed by self-assembly into a nanoparticle with free hydroxycamptothecin, (**B**) chemical structure of the CMC copolymer, and (**C**) TEM micrographs of the core-shell CMC-based nanoparticles. Reproduced with permission from [159]. Copyright Royal Society of Chemistry, 2015.

In the case of cellulose derivatives, they are usually dissolved in organic solvents like DMSO or ethanol, which are then removed via evaporation, obtaining stable aqueous dispersions of the corresponding nanoparticles [159,161]. For example, Dai et al. [159] reported the preparation of CMC nanoparticles through the synthesis of a CMC derivative (betulinic acid and PEGylated-folate grafted into the CMC polymer chain), followed by self-assembly into a nanoparticle with free hydroxycamptothecin, which is an anticancer drug (Figure 5A,B). The core-shell CMC nanoparticles were self-assembled via the slow addition of a DMSO solution of the CMC derivative into aqueous media (Figure 5C). The variation of the concentration of the CMC derivative in the organic media (20 to 100 mg mL^−1^) originated nanoparticles with distinct sizes, namely the particles formed from 50 mg mL^−1^ solutions were approximately 150 nm in size, while the particles formed from 20 mg mL^−1^ solutions were smaller (90–110 nm) [159].

### 5.2. Emulsification Processes

Emulsification processes can also be used to manufacture sphere-shaped cellulose-based nanoparticles. As in the case of cellulose-based microparticles (Section 4.1), cellulose derivatives, such as CAP [167], CMC [168], and EC [141,169,170,171,172,173,185], are widely studied as the cellulosic substrate to engineer sphere-shaped cellulose-based nanoparticles, as enumerated in Table 5, either in the form of nanospheres [167] or nanocapsules [141,167]. Although out of the scope of the present review, it is worth mentioning that some of the nanoscale forms of cellulose, namely CNCs and CNFs, are also being used as emulsion stabilizers in the synthesis of polymeric nanoparticles [188,189,190,191,192].

A representative example of nanoparticles assembled via emulsification processes includes the work of Vidal-Romero et al. [167], who developed pH-dependent systems based on nanospheres and nanocapsules of CAP loaded with chlorhexidine as a novel formulation for periodontal treatment. The nanospheres and nanocapsules were prepared by emulsion-diffusion technique, where the presence of eugenol oil originated CAP nanocapsules (diameter: 290–324 nm) and its absence yielded CAP nanospheres (diameter: ~248 nm), due to the effect of the oil on the interfacial behaviour during the formation process, viz. diffusion-stranding mechanism [167]. The superior size of the CAP nanocapsules, when compared with the CAP nanospheres, is also credited to the presence of eugenol oil.

The investigation of Abbaspoor et al. [141] reported on the effect of the stirring rate during the emulsification-solvent evaporation method on the production of self-healing coatings [185] based on nanocapsules of EC as the shell and linseed oil as the core material. The size of the oil-filled EC nanocapsules can be successfully modulated by controlling the emulsion stirring rate (10,000, 20,000 and 30,000 rpm) during the production process, decreasing from 472.8 nm to 32.9 nm by increasing the stirring rate from 10,000 to 30,000 rpm [141].

In a different study, Tirado et al. [173] used the supercritical emulsion extraction (SEE) technology, which associates emulsification processes with the singular properties of supercritical fluids, to prepare EC nanocapsules for the encapsulation of bioactive compounds. The authors studied distinct emulsion formulations by varying the EC concentration (1.0, 1.5, 2.0 and 2.5 wt.%), and surfactant amount (0.1, 0.2, 0.3 and 0.6 wt.%), both of which influenced the morphology and size of the ensuing nanoparticles.

### 5.3. Other Methodologies

In addition to nanoprecipitation and emulsification, there are other less used methodologies, such as mechanical treatments [174,175,176,177], chemical and/or enzymatic treatments [178,179,180,181,182], self-assembly processes [193,194,195,196,197,198,199,200], and layer-by-layer assembly [183], which can be applied (individually or in combination) to manufacture cellulose-based nanoparticles (Table 6).

The mechanical methods reduce the size of cellulose fibres via several passages of the pulp suspension through the system [201]. Even though these processes are commonly applied to obtain nanofibrils, some works also describe the obtention of spherical cellulose particles (Table 6). In a set of experiments performed by Yan et al. [174,175], high-pressure homogenization of bamboo pulp was combined with periodate oxidation. Mechanical treatment allowed the size reduction of bamboo, after which the cellulosic pulp was subjected to a periodate oxidation to yield dialdehyde cellulose (DAC). After the optimization of the experimental conditions, the authors were able to generate cellulose nanoparticles with sizes from 15 to 35 nm by aging the DAC solution for 10 days at 60 °C [175].

The application of an ultrasonic treatment, resulting in the cavitation of cellulosic solutions, has also been reported for the preparation of cellulose-based nanoparticles. For instance, TEMPO-modified cellulose was subjected to sonication, converting cellulose into nanoparticles with sizes below 30 nm. These were further oxidized in the presence of sodium periodate to obtain cellulose nanoparticles decorated with functional groups, allowing linkage with other molecules (e.g., for the controlled delivery of drugs) [176].

In the domain of chemical and/or enzymatic treatments (Table 6), Chen et al. [179] reported the preparation of ca. 30 nm-sized cellulose nanoparticles through enzymatic hydrolysis of eucalyptus pulp. Based on preliminary studies, a mixture of xylanase/cellulase was chosen for the enzymatic breakdown of cellulose. Using specific well-defined conditions, particularly the concentration of enzymes (ratio of cellulase to xylanase, 9:1), reaction temperature (50 °C) and time (5 h), spherical cellulose nanocrystals were successfully obtained. The decrease of reaction time (from some days to a mere couple of hours) was the main highpoint of this work, and the same set of conditions was later replicated in another study with similar results [180].

The studies regarding the use of acid hydrolysis usually employ a combination of two acids, either hydrochloric and sulfuric acids [178] or hydrochloric and formic acids [181,182]. The procedures usually start with a pre-treatment stage, using a NaOH aqueous solution to remove impurities and to swell the amorphous regions of the cellulose fibres. The resultant suspensions are subjected to acid hydrolysis, after which they are also exposed to ultrasonication to induce acoustic cavitation effect (growth and collapse of bubbles under ultrasonic irradiation) to generate particles [202]. The impact of the proportion of the acids mixtures in this hydrolysis procedure was observed, for instance, in the size of the particles obtained by Yan et al. [181]. In this study, smaller sizes and a narrower size distribution were attributed to the higher ratio (9:1) of hydrochloric and formic acids and highest hydrolysis period (8 h). The authors also reported a reduction in nanoparticle size with the decrease of fibre/acid mixture ratio (smallest particles with ca. 19 nm) [181].

In another work, spherical cellulose nanocrystals obtained from cotton linter powder [178] were bigger (45–75 nm) than those obtained from lyocell fibres (under 30 nm) [181,182], suggesting that the choice of the starting material also plays a role on particle size. Interestingly, the alteration of the starting material from lyocell fibres to microcrystalline cellulose (and simultaneous reduction in hydrolysis time from 8 h to 4 h) resulted in rod-shaped cellulose nanocrystals, highlighting the need for a delicate balance in reaction conditions to obtain the sought-after spheres [182].

Cellulose-based nanoparticles can also be prepared by self-assembly methodologies through the synthesis of cellulose derived copolymers, mostly from cellulose derivatives as the starting cellulosic substrate (Table 6). For instance, the grafting polymerization of CMC with dimethyldiallylammonium chloride resulted in CMC-*g*-PDMDAAC copolymer particles of 100–150 nm and asymmetrical shape [193]. Upon loading of the avermectin pesticide, the particles revealed a size of 120–180 nm and a spherical form, as the negatively-charged avermectin balanced the internal charge and resulted in a capsule-like structure that encased the drug [193]. The choice of the comonomer has proven to impact the properties of the ensuing particles. As pointed out by Chen et al. [194], the grafting of CMC with either styrene (S), methyl methacrylate (MMA) or butyl acrylate (BA), originated nanoparticles with distinct size, with CMC-*g*-PBA yielding the smallest particles (*ca*. 180 nm). Interestingly, this also affected the drug loading (higher for MMA and BA) and drug-release speed (CMC-*g*-PBA > CMC-*g*-PS > CMC-*g*-PMMA) [194].

Interestingly, some copolymers are amphiphilic in nature and self-assemble into spherical micellar structures in aqueous media. An example of this are the di-block copolymer micelles with 175–216 nm, synthesized by Lu et al. [200] from hydroxypropyl methyl cellulose (HPMC) and poly(lactic acid) (PLA) for drug delivery. Equally interesting is the host–guest driven self-assembly strategy, where cellulose-based nanoparticles can be assembled via host–guest molecular recognition. For example, Yang et al. [199] generated nanoparticles with diameters of ca. 36 nm through the interaction of adamantane-grafted carboxyethyl hydroxyethyl cellulose (CEHEC-*g*-Ad) with β-cyclodextrin-grafted glycerol ethoxylate (GE-CD), as guest and host polymers, respectively.

**Figure 6:**
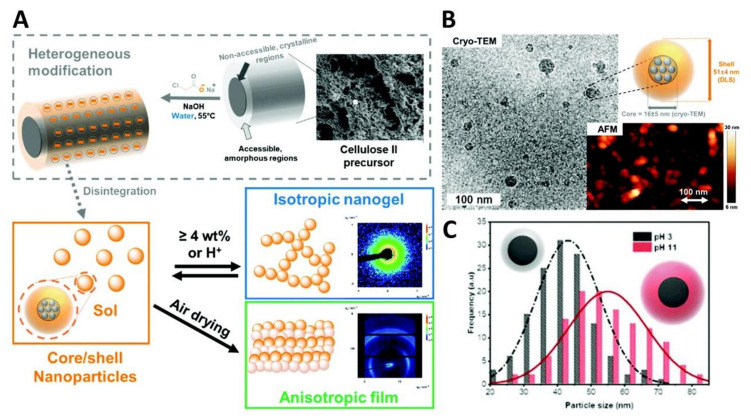
Schematic representation of (**A**) the synthesis of the core-shell cellulose nanoparticles from a cellulose II precursor via heterogeneous modification by carboxymethylation followed by disintegration, (**B**) morphology and size of the anionic cellulose II nanoparticles studied by cryo-transmission electron microscopy (TEM), atomic force microscopy (AFM) and dynamic light scattering (DLS), and (**C**) particle size distribution at pH 3 and 11 from AFM analysis. Reproduced with permission from [204]. Copyright Royal Society of Chemistry, 2019.

Beaumont et al. [204] assembled soft cellulose nanoparticles via organic solvent-free heterogeneous modification followed by disintegration (Figure 6A). The simple synthesis of anionic carboxylated cellulose from a commercial regenerated cellulose precursor (LENZING™ Lyocell fibres) originated nanoparticles bearing a semi-crystalline core (16 ± 5 nm determined by TEM) and a distinctive amorphous outer shell structure (51 ± 4 nm determined by DLS), as depicted in Figure 6B. Furthermore, the authors studied the sol-gel behaviour of these core/shell nanoparticles and observed that (i) the particle size distribution varied with ionic strength and pH (Figure 6C), and (ii) the supercritical drying of the hydrogels originated an isotropic and highly porous aerogel of aggregated nanoparticles, whereas ambient drying yielded an anisotropic and fully transparent film (Figure 6A).

Other noteworthy results were reported by Elumalai et al. [183], who prepared hollow nanocapsules with bilayers of CMC and protamine with ca. 150 nm through layer-by-layer assembly (Table 6) around a sacrificial silica template. The deposition of three bilayers was achieved, with protamine as the outer layer, and the resulting capsules were later modified with magnetic Fe_3_O_4_ nanoparticles, aiming to enhance drug delivery of anticancer drugs in the presence of a magnetic field [183].

## 6. Applications of Spherical Cellulose-Based Micro and Nanoparticles

The versatility of cellulose grants researchers with unending possibilities of novel sphere-shaped micro and nanoparticles with diverse applications, as evidenced in Table 1, Table 2, Table 3, Table 4, Table 5, Table 6, spanning from drug delivery [126,130,149,152,166,168] to cell culture and tissue engineering [113,116,117,118,144], as well as water remediation [115,119,136,148,178,207,208],. The following paragraphs explore some recent examples of applications of the micro and nanoparticles engineered from cellulose and derivatives thereof.

A considerable amount of the work carried out with cellulose-based sphere-shaped particles envisions the development of new systems for drug delivery, mostly based on nano-sized particles produced by nanoprecipitation (Table 4) and emulsification (Table 5) processes. As an example, oxidized cellulose [176] and other derivatives like hydroxypropyl methyl cellulose [200] and hydroxyethyl cellulose [199] have been applied for the encapsulation of anticancer drugs, such as doxorubicin [176,183], 5-fluorouracil [127], and paclitaxel [200]. However, the encapsulation of drugs is not restricted to anticancer drugs. In fact, antiretroviral medication (e.g., zidovudine [168]), antibiotics (e.g., penicillin G [166] and tetracycline [131]), anti-inflammatory drugs (e.g., diclofenac [134], ibuprofen [209], ketoprofen lysinate [126], and naproxen [123]), vaccines (e.g., foot-and-mouth disease virus (FMDV) subunit vaccine [147]), antiseptics (e.g., chlorhexidine [167]), pesticides (e.g., emamectin benzoate [146] and avermectin [193]), and other bioactive molecules or model drugs (e.g., dexamethasone [172], curcumin [160], astaxanthin [173], α-mangostin [170], hydroxytyrosol [153], and aminoethyl rhodamine [164]), have also been successfully encapsulated in cellulose-based particles to treat other ailments. As a representative example, EC nanoparticles were prepared by El-Habashy et al. [171] to modulate the release profile of piroxicam to reduce the ulcerogenicity of this anti-inflammatory drug after oral administration. The encapsulation of this molecule significantly reduced the gastric ulceration potential of piroxicam in rats, with a reduction of 66% in mean ulcer index.

Additionally, sphere-shaped micro and nanoparticles based on cellulose, or its derivatives, are also largely used in cell culture and tissue engineering due to the innocuous nature (causing no cell-death upon exposure) of this polysaccharide while possessing notable mechanical properties. In fact, there are even commercially available cellulose-based microparticles for cell culture [66], like the Cytopore™ macroporous microcarriers composed of 100% cotton cellulose particles with diameters of 200 to 280 μm (Cytiva™ technologies) [35]. Herein, most of the particles are micro-sized and fabricated by emulsification (Table 1) and microfluidics (Table 2). In terms of cells, the cellulose-based particles have been explored for the cell culture of several animal and human cell lines, such as mouse NIH 3T3 cells [142], murine osteoblast (OB-6) cells [210], bone marrow-derived mesenchymal stem cells (BMSCs) [144], mouse MC3T3-E1 cells [113], human adenocarcinoma from lung tissue PC-9 cells [116], and human liver carcinoma HepG2 cells [120].

**Figure 7:**
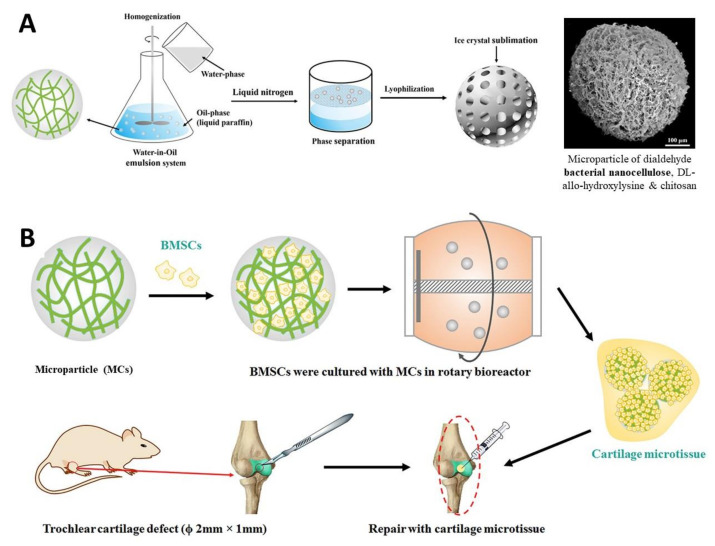
Schematic illustration of (**A**) the fabrication procedure and SEM micrograph of the microparticles composed of dialdehyde bacterial nanocellulose, DL-allo-hydroxylysine and chitosan, and (**B**) the overall in vitro and in vivo experiments design with the bone marrow-derived mesenchymal stem cells (BMSCs) and regeneration of the knee articular cartilage defect in mice. Reproduced with permission from [144]. Copyright Elsevier, 2018.

As an illustrative example, Wang et al. [144] developed microparticles (~450 μm) based on BNC, DL-allo-hydroxylysine and chitosan via emulsification (Figure 7A), to mimic the natural extracellular matrix. The resulting particles promoted the in vitro cell growth and proliferation of BMSCs cells (Figure 7B). A cartilage microtissue, obtained from BMSCs cultured in these microparticles, was used for the in vivo regeneration of a knee articular cartilage defect in mice, showing no immunological complications and contributing to the cartilage regeneration. The authors stated that the mechanical features of the repaired tissues are analogous to those of normal cartilage [144].

In the field of water remediation, many recent works focus on the use of cellulose-based particles for the adsorption and removal of metal ions (e.g., Hg^2+^ [178], Eu^3+^ [208], Cu^2+^ [129,207,211], Cd^2+^ [211], and Pb^2+^ [211,212]), metal nanoparticles (e.g., silver and gold nanoparticles [151]), and organic dyes (e.g., methylene blue [136,148], rhodamine 6G [148], Congo red [119], crystal violet, and methyl orange [115]). Here, the majority of the particles are micro-sized, and manufactured by emulsification processes (Table 1) from cellulose derivatives.

**Figure 8:**
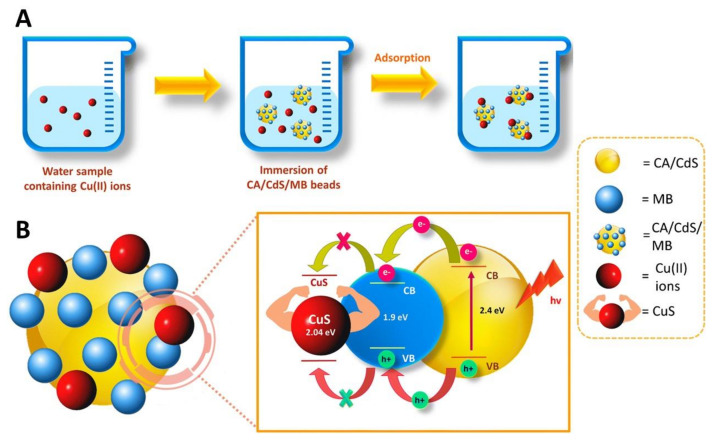
Schematic representation of the (**A**) adsorption of Cu(II) ions on the surface of the hybrid cellulose acetate microparticles decorated with cadmium sulphide (CdS) and methylene blue (MB), and (**B**) stepwise electron transfer for photoelectrochemical detection of Cu(II) ions under visible light radiation. Reproduced with permission from [207]. Copyright Springer, 2019.

Ibrahim et al. [207] fabricated multi-functional hybrid cellulose acetate microparticles (diameter of 684 μm) decorated with cadmium sulphide and methylene blue (CA/CdS/MB, Figure 8A), and investigated their application as a photosensor-adsorbent for the rapid, selective and sensitive detection, and adsorption of Cu(II) ions (Figure 8B). The CA derived microparticle photosensor-adsorbent showed an adsorption capacity of 0.57 mg g^−1^ in the photoelectrochemical detection of Cu(II) ions.

On a different approach, Park et al. [115] developed cellulose/biopolymer/Fe_3_O_4_ particles (*ca.* 60 nm) to remove crystal violet and methyl orange from contaminated waters. The results differed whether the cellulose particles were blended with chitosan or κ-carrageenan. Cellulose/carrageenan/Fe_3_O_4_ microparticles showed a 1.3-fold higher adsorption of crystal violet, while the cellulose/chitosan/Fe_3_O_4_ revealed a better performance (2.0 times higher) for methyl orange (in both cases, when compared with the cellulose counterparts). This is justified by the particles surface charge since the electrostatic attraction between the positively charged crystal violet and the sulphate groups of κ-carrageenan favoured the adsorption of the dye. Similarly, the amino groups of chitosan may have caused an analogous effect on the negatively charged methyl orange dye [115].

Other application fields for sphere-shaped cellulose-based particles encompass coatings [184,185], functional textiles [114] and transistors and batteries [162], but also the commercial examples of cellulose microparticles, namely the Cellufine™, viz. cellulose spherical beads with particle size of ca. 40–130 µm, used as chromatography media [33], and the Macroporous Bead Cellulose MT, i.e., highly porous regenerated cellulose with particle size of ca. 30–250 µm, utilized as gel filtration media for biomolecule separations [34].

## 7. Conclusions and Future Directions

Given the increasing concerns with sustainability [21], the dawn of a renewable and easily available natural raw-material, such as cellulose, is welcomed in many fields of modern science and technology. Cellulose is undeniably a natural polymer of notorious versatility, which can be easily functionalized and combined with other molecules and macromolecules to allow countless possibilities for particle engineering. However, its insolubility in water and in most conventional solvents remains as the most challenging aspect of cellulose processing. Nonetheless, new solvent alternatives (e.g., ionic liquids and deep eutectic solvents [71], switchable solvents [87] or organic electrolyte solutions [88,89]) for cellulose green and safe dissolution and chemical conversion, have emerged to circumvent this constraint. Still, cellulose derivatives (e.g., carboxymethylcellulose, cellulose acetate, and ethyl cellulose) continue to be at the forefront of cellulose-based spherical particle research, due to their straightforward processability.

Regarding the available manufacturing strategies, conventional approaches, like emulsification processes (Table 1 and Table 5) and nanoprecipitation (Table 4), continue to be the most explored for the preparation of cellulose-based particles, given their simplicity and cost-effectiveness. The emulsification processes are suitable to produce both spheres and capsules at the micro and nanoscale ranges (Table 1 and Table 5, respectively), while the nanoprecipitation is a methodology commonly used to fabricate nanoparticles (Table 4), as an alternative to the emulsion process. Other methods are also gaining increasing attention, such as the microfluidic technology (Table 2), due to the facile tunability of particle features. Additional strategies include layer-by-layer assembly and spray-assisted techniques for microparticles production (Table 3), and mechanical, chemical and enzymatic treatments, self-assembly processes, and layer-by-layer assembly for nanoparticles fabrication (Table 6). The precise control of process parameters dictates the shape and size, as well as surface chemistry of the resulting particles. For example, the stirring rate and surfactant type are two of the most important parameters in emulsification processes, responsible for generating particles either at the micro or nanoscale ranges. In the case of the microfluidics technology, the type of device and the flow rate of the continuous and dispersed phases are crucial parameters to adjust the size and shape of the ensuing cellulose-based microparticles. Regarding nanoprecipitation, the concentration of cellulose or its derivatives and the solvent/antisolvent ratio deeply influence the size of the cellulose-based nanoparticles.

In terms of scalability, it is already a reality for niche markets in the case of cellulose microparticles, such as the Cellufine™ chromatography media for the purification of proteins, enzymes, and other biomolecules (JNC Corporation) [33], the Macroporous Bead Cellulose MT for application as gel filtration media for biomolecule separations (IONTOSORB^®^ Company) [34], and the Cytopore™ macroporous microcarriers for use in stirred suspension culture systems for the growth of cells and the production of recombinant proteins for therapeutic use, as well as for the immobilization of insect cells, yeast, and bacteria (Cytiva™ technologies) [35]. On the contrary, there are several key obstacles related with the downscale of the cellulose (or derivatives thereof) shaping into nanoparticles via dissolution and coagulation, together with the lack of regulatory guidance for their safe use and disposal, that are delaying the commercial translation of the majority of the examples of the sphere-shaped cellulose-based nanoparticles portrayed in the present appraisal.

Overall, there is a fast-growing tendency for researchers to develop particles for biomedical applications, given the enduring need for novel and efficient healthcare solutions in the field of drug delivery, cell culture, and tissue engineering. In fact, the remarkable characteristics of cellulose are potentiating interesting developments on the controlled delivery of drugs and bioactive molecules. Nevertheless, the variety of particles described here results in an increasing impact in many fields, for instance, the growing concern with water-remediation has also driven a lot of research into cellulose-based solutions.

The relevance of this topic is proven by the number of recent works focused on exploiting conventional and new approaches for the use of cellulose and cellulose-derivatives as an unmatched family of versatile and sustainable materials for particle fabrication. We foresee that the sphere-shaped cellulose-based micro and nanoparticles will be produced more efficiently and at a lower cost with the development of new production technologies or the improvement of the existing ones, as well as the utilization of greener and more efficient solvent systems for cellulose dissolution and regeneration. Furthermore, the spherical cellulose-based micro and nanoparticles will continue to run the gamut of applications from medicine, biology and environment to electronics and energy.

## Figures and Tables

**Table 1:** Examples of spherical cellulose-based microparticles produced by emulsification processes.

Cellulosic Substrate	General Features	Diameter (μm)	Application	Ref.
Bamboo pulp	*Aqueous phase*: cellulose in NaOH/urea/H_2_O (7:12:81 wt.%) *Oil phase*: paraffin oil/Span^®^ 80 *Emulsion type*: *w*/*o* Modified with magnetic nanoparticles and poly(DOPAm-*co*-PFOEA) *a posteriori*	~30 (microcapsule)	–	[112]
BNC	*Aqueous phase*: gelatin/*K. xylinus* bacterium *Oil phase*: corn oil/Span^®^ 80 *Emulsion type*: *w*/*o*	~10 (microsphere)	–	[117]
BNC	*Aqueous phase*: oxidized BNC in [C1mim][Cl]/collagen/polystyrene templates/TWEEN^®^ 80 *Oil phase*: n-hexadecane/Span^®^ 80 *Emulsion type*: *w*/*o*	8–12 (microsphere)	Cell culture (MC3T3-E1 cells) Drug delivery (model drug: BSA)	[113]
BNC	*Aqueous phase*: DHYL-DBC/chitosan in acetic acid solution *Oil phase*: Paraffin oil *Emulsion type*: *w*/*o*	~450 (microsphere)	Cell culture (BMSCs cells)	[144]
BNC	*Aqueous phase*: 2% *v*/*v* bacterial solution *Oil phase*: decane *Emulsion type*: *w*/*o*	350 (microcapsule)	–	[125]
CA	*Aqueous phase*: PVA in water *Oil phase*: CA in ethyl acetate/eugenol *Emulsion type*: *o*/*w*	~1.3 (microsphere)	Functional textiles (active agent: eugenol)	[114]
CA	*Aqueous phase*: Span^®^ 80/TWEEN^®^ 80 in water *Oil phase*: CA in DMF/DCM *Emulsion type*: *w*/*o*	~5 (microsphere)	Catalysis	[145]
CAB	*Aqueous phase*: PVA in water *Oil phase*: CAB in chloroform/EB *Emulsion type*: *o*/*w*	70–150 (microsphere)	Pesticide delivery (EB)	[146]
CAB	*Aqueous phase*: PVA in water *Oil phase*: CAB in ethyl acetate/eugenol *Emulsion type*: *o*/*w*	~2.2 (microsphere)	Functional textiles (active agent: eugenol)	[114]
CAP	*Aqueous phase*: PVA in water *Oil phase*: CAP in chloroform + ethanol/eugenol *Emulsion type*: *o*/*w*	~20 (microsphere)	Functional textiles (active agent: eugenol)	[114]
CAP (thiolated)	*Aqueous phase*: w1: M5BT/Pluronic^®^ F-127, w2: PVA in water *Oil phase*: CAP in DCM and ethyl acetate/ethanol *Emulsion type*: *w*/*o*/*w*	~3.20 (microsphere)	Oral vaccination (M5BT subunit vaccine)	[147]
CNFs	*Aqueous phase*: CNFs/PVA in deionized water/glutaraldehyde *Oil phase*: Toluene/Span^®^ 80 *Emulsion type*: *w*/*o* (Crosslinking with glutaraldehyde)	94.5 ± 16.1 503.9 ± 73.5 (microsphere)	Cell culture (NIH 3T3 cells)	[142]
Cotton linter pulp	*Aqueous phase*: cotton pulp dissolved in NaOH/zinc nitrate aqueous solution *Oil phase*: Isooctane/Span^®^ 80 *Emulsion type*: *w*/*o* (*In situ* precipitation of ZnO nanoparticles)	~60 (microsphere)	–	[135]
Cotton linter pulp	*Aqueous phase*: cellulose in NaOH/urea/ H_2_O + tannins from *Areca catechu* *Oil phase*: Paraffin oil/Span^®^ 80/TWEEN^®^ 80 *Emulsion type*: *w*/*o* (Microcapsules crosslinked with epichlorohydrin)	408 ± 15 (microsphere)	Water remediation (organic dye: methylene blue)	[136]
Cotton linter pulp	*Aqueous phase*: Cellulose in NaOH/urea/H_2_O *Oil phase*: Paraffin oil/Span^®^ 80 *Emulsion type*: *w*/*o*	~12 (microsphere)	HILIC stationary phase	[137]
CP	*Aqueous phase*: CP in NaOH solution *Oil phase*: 1,2-dichloroethane with CAB *Emulsion type*: *w*/*o* (Microsphere crosslinked with epichlorohydrin)	10–20 (microsphere)	Water remediation (organic dyes: methylene blue, rhodamine 6G)	[148]
CS	*Aqueous phase*: CS/alginate/chitosan *Oil phase*: Isooctane/Span^®^ 80+TWEEN^®^ 80 *Emulsion type*: *w*/*o*	62.4 ± 13.9 (microcapsule)	–	[149]
EC	*Aqueous phase*: PEG/SDS in nitric acid aqueous solution *Oil phase*: EC in benzene+ ethanol/linseed oil *Emulsion type*: *o*/*w*	400 (microcapsule)	–	[141]
EC	*Aqueous phase*: methylcellulose in water *Oil phase*: EC in chloroform/Pheo-a *Emulsion type*: *o*/*w*	149–163 (microsphere)	–	[133]
EC	*Aqueous phase*: glycerin in water/PVA *Oil phase*: EC in acetone *Emulsion type*: *o*/*w*	13.7 ± 0.5 (microsphere)	–	[140]
EC	*Aqueous phase*: w1: water, w2: PVA in water *Oil phase*: EC in DCM or DCM/methanol or chloroform or ethyl acetate *Emulsion type*: *w*/*o*/*w*	60–133 (microsphere)	–	[150]
EC	*Aqueous phase*: w1: PVA in water, w2: PEI in water *Oil phase*: EC in chloroform/PVP/SDS *Emulsion type*: *w*/*o*/*w* (Microcapsules crosslinked with glutaraldehyde)	5–30 (microcapsule)	Water remediation (metal nanoparticles: Ag and Au NPs)	[151]
EC	*Aqueous phase*: PVA in water *Oil phase*: EC in chloroform+ ethanol/eugenol *Emulsion type*: *o*/*w*	~11.5 (microsphere)	Functional textiles (active agent: eugenol)	[114]
EC	*Aqueous phase*: Distilled water/TWEEN^®^ 80 *Oil phase*: EC in ethyl acetate or ethyl acetate/acetone *Emulsion type*: *o*/*w*	382.4 ± 0.6 to 998.1 ± 0.8 (microsphere)	Drug delivery (NSAID: diclofenac)	[134]
EC	*Aqueous phase*: sodium bicarbonate aqueous solution *Oil phase*: o1: EC in acetonitrile/TWEEN^®^ 80 o2: Soybean oil/Span^®^ 80 *Emulsion type*: *s/o/o*	280–340 (microsphere)	Drug delivery (model drug: sodium bicarbonate)	[152]
EC	*Aqueous phase*: w1: hydroxytyrosol in double distilled water, w2: PVA in water *Oil phase*: EC in DCM *Emulsion type*: *w*/*o*/w	156.6 ± 6.9 to 304.0 ± 16.0 (microcapsule)	Drug release (model drug: hydroxytyrosol)	[153]
MCC	*Aqueous phase*: MCC/Fe_3_O_4_/chitosan, κ-carrageenan, lignin or starch in [C2mim][Ac] *Oil phase*: Pump oil/Span^®^ 80 *Emulsion type*: *w*/*o*	39–62 (microsphere)	Protein immobilization (pepsin, BSA, lysozyme) Water remediation (organic dyes: crystal violet, methyl orange)	[115]
MCC	*Aqueous phase*: MCC in NaOH/urea/H_2_O *Oil phase*: o1: cellulose solution/paraffin oil, o2: nonsolvent+Span^®^ 80/paraffin oil *Emulsion type*: *w*/*o*/o	5.4 ± 1.8 to 20.9 ± 8.9 (microsphere)	–	[93]
MCC	*Dispersed phase*: MCC in [C2mim][Ac]/ DMSO *Continuous phase*: sunflower oil/Span^®^ 80 *Emulsion type*: *w*/*o*	17–135 (microsphere)	–	[138]
MCC	*Dispersed phase*: MCC/[Bmim]Cl/DMF *Continuous phase*: cyclohexane/Hypermer 1599™ + TWEEN^®^ 80 *Emulsion type*: o/o	23 ± 19 to 54 ± 36 (microsphere)	Drug delivery (analgesic drug: acetaminophen)	[143]

Abbreviations: AZT: zidovudine; [Bmim]Cl: 1-butyl-3-methylimidazolium chloride; BMSCs: bone marrow-derived mesenchymal stem cells; BNC: bacterial nanocellulose; BSA: bovine serum albumin; [C_1_mim][Cl]: 1-allyl-3-methylimidazolium chloride; [C_2_mim][Ac]: 1-ethyl-3-methylimidazolium acetate; CA: cellulose acetate; CAB: cellulose acetate butyrate; CAP: cellulose acetate phthalate; CNFs: cellulose nanofibrils; CP: cellulose phosphate; CS: cellulose sulphate; DCM: dichloromethane; DHYL-DBC: DL-allo-hydroxylysine grafted C2, 3-dialdehyde bacterial cellulose; DMF: *N*,*N*-dimethylformamide; DMSO: dimethyl sulfoxide; EB: emamectin benzoate; EC: ethyl cellulose; HILIC: hydrophilic interaction liquid chromatography; Hypermer 1599™: oil soluble polymeric ester surfactant; M5BT: multi-epitope recombinant protein derived from virus from foot-and-mouth disease; MCC: microcrystalline cellulose; MC3T3-E1: osteoblast precursor cell line derived from *Mus musculus* (mouse) calvaria; NIH 3T3: Swiss mouse fibroblast embryo cell line; NPs: nanoparticles; NSAID: nonsteroidal anti-inflammatory drug; o1: primary oil-phase; o2: secondary oil-phase; o/o: oil-in-oil; *o*/*w*: oil-in-water; PEG: poly(ethylene glycol); PEI: poly(ethylenimine); Pheo-a: pheophytin-a; Pluronic^®^ F-127: triblock copolymer of poly(ethylene oxide) and poly(propylene oxide) (non-ionic surfactant); poly(DOPAm-*co*-PFOEA): *N*-(3,4-dihydroxyphenethyl) acrylamide-2-perfluorooctyl)ethyl acrylate copolymer; PVA: poly(vinyl alcohol); PVP: poly(vinylpyrrolidone); s/o/o: solid-in-oil-in-oil; SDS: Sodium dodecyl sulphate; Span^®^ 80: sorbitan monooleate (non-ionic surfactant); TWEEN^®^ 80: polyethylene glycol sorbitan monooleate (non-ionic surfactant); w1: primary aqueous-phase; w2: secondary aqueous-phase; *w*/*o*/o: water-in-oil-in-oil; *w*/*o*/w: water-in-oil-in-water; *w*/*o*: water-in-oil.

**Table 2:** Examples of spherical cellulose-based microparticles produced by microfluidics.

Cellulosic Substrate	General Features	Diameter (μm)	Application	Ref.
BNC	*Dispersed phase*: alginate microcapsules/agarose/*G. xylinus*/culture medium *Continuous phase*: HFE-7500 fluorocarbon oil/ Krytox™ modified with PEG *Q*_D_ = 0.1–0.5 µL min^−1^; *Q*_C_ = 5 µL min^−1^ Cross-junction droplet generator	~50 (microcapsule)	Cell culture (PC-9 cells) Wound healing (rat skin model)	[116]
BNC	*Dispersed phase*: gelatin + bacteria *Continuous phase*: corn oil with Span^®^ 80 *Q*_D_ = NR; *Q*_C_ = 50–1000 µL min^−1^ Co-flow microfluidic device	~250–1000 (microsphere)	–	[117]
BNC	*Dispersed phase*: *A. xylinum*/ culture medium *Continuous phase*: hydrogenated castor oil *Q*_D_ = 1.2 µL min^−1^; *Q*_C_ = 12 µL min^−1^ Co-flow microfluidic device	>100 (microcapsule)	–	[118]
BNC	*Dispersed phase*: pure medium (inner phase) and bacterial suspension (middle phase) *Continuous phase*: decane with surfactant (Span^®^ 85 or phosphatidylcholine) *Q*_D_ = 200 μL h^−1^ (inner phase) and 800 μL h^−1^ (middle phase); *Q*_C_ = ~333 µL min^−1^ Flow-focusing device for transient double emulsions	80−500 (microcapsule)	–	[125]
CA	*Dispersed phase*: CA in DMA, DMF or DMSO *Continuous phase*: n-hexane/ Span^®^ 80 *Q*_D_ = 10 µL min^−1^; *Q*_C_ = 100–400 µL min^−1^ T-junction microfluidic device	270–750 (microsphere)	Water remediation (organic dye: Congo red)	[119]
CMC	*Dispersed phase*: Ph-CMC/DEX/HRP *Continuous phase*: PEG/PEG and H_2_O_2_ *Q*_D_ = NR; *Q*_C_ = NR Co-flow microfluidic device	65–111 (microcapsule)	Cell culture (HepG2 cells)	[120]
CNCs	*Dispersed phase*: sCNCs or aCNCs/hCNCs *Continuous phase*: soybean oil/PGPR *Q*_D_ = 1.6–4 µL min^−1^; *Q*_C_ = 2–5 µL min^−1^ T-junction droplet microfluidic device	30–110 (microcapsule)	–	[121]
CNFs	*Dispersed phase*: CNFs water suspension *Continuous phase*: MADQUAT-*co*-BTA in toluene *Q*_D_ = NR; *Q*_C_ = NR Glass capillary microfluidic device	303 ± 3.4 (microcapsule)	–	[122]
CNFs (TEMPO oxidized)	*Dispersed phase*: aqueous CNFs suspension *Continuous phase*: oleylamine/toluene solution *Q*_D_ = 10−40 µL min^−1^; *Q*_C_ = 200−400 µL min^−1^ T-junction microfluidic device	25–200 (microcapsule)	–	[155]
Dissolving cellulose pulp	*Dispersed phase*: octane (inner phase) and cellulose solution of LiCl/DMA (middle phase) *Continuous phase*: silicone oil *Q*_D_ = 10 μL h^−1^ (inner phase) and 60 μL h^−1^ (middle phase); *Q*_C_ = 2,000 μL h^−1^ Microfluidic flow focusing device	88 μm (microcapsule)	Drug delivery (model drug: FITC-dextran)	[156]
EC	*Dispersed phase*: EC/ROY or EC/carbamazepine in dichloromethane *Continuous phase*: aqueous PVA solution *Q*_D_ = NR; *Q*_C_ = NR Glass capillary microfluidic device (counter-flow configuration)	150−300 (microsphere)	Drug delivery (model drug: ROY; anticonvulsant drug: carbamazepine)	[124]
EC	*Dispersed phase*: EC/naproxen in ethyl acetate *Continuous phase*: aqueous PVA solution *Q*_D_ = 200−500 µL min^−1^; *Q*_C_ = 50−120 µL min^−1^ Microfluidic T-junction device	55–220 (microsphere)	Drug delivery (NSAID: naproxen)	[123]

Abbreviations: aCNCs: aldehyde-modified cellulose nanocrystals; BNC: bacterial nanocellulose; CA: cellulose acetate; CMC: carboxymethylcellulose; CNCs: cellulose nanocrystals; CNFs: cellulose nanofibrils; DEX: dextran; DMA: dimethylacetamide; DMF: *N*,*N*-dimethylformamide; DMSO: dimethyl sulfoxide; EC: ethyl cellulose; FITC: fluorescein isothiocyanate; hCNCs: hydrazide-modified CNCs; HepG2: human liver carcinoma cell line; HRP: horseradish peroxidase; MADQUAT-*co*-BTA: [2-(methacryloyloxy)ethyl]-trimethylammonium chloride-*co*-butyl acrylate copolymer; NR: not reported; NSAID: nonsteroidal anti-inflammatory drug; PC-9: human lung adenocarcinoma cell line; PEG: (polyethylene)glycol; PGPR: polyglycerol polyricinoleate; Ph-CMC: phenolic modified CMC; PVA: poly(vinyl alcohol); Q_C_: flow rate of the continuous phase; Q_D_: flow rate of the dispersed phase; ROY: 5-methyl-2-[(2-nitrophenyl)amino]-3-thiophenecarbonitrile; sCNCs: sulphated CNCs; Span^®^ 80: sorbitan monooleate (non-ionic surfactant); Span^®^ 85: sorbitan trioleate (non-ionic surfactant).

**Table 3:** Examples of spherical cellulose-based microparticles produced by other methodologies.

Cellulosic Substrate	General Features	Diameter (μm)	Application	Ref.
LAYER-BY-LAYER ASSEMBLY				
CMC	*Methodology*: (CMC/CH)_16_ bilayers on a MF template	~2.15 (microcapsule)	Drug delivery (antibiotic: tetracycline)	[131]
QA-CNFs	*Methodology*: (CNFs/XyG/CNFs/AP)_2_CNFs /XyG bilayers on a CaCO_3_ template	16 ± 4 (microcapsule)	Drug delivery Cell culture (HEK 293T cells)	[132]
SPRAY-ASSISTED TECHNIQUES				
CA	*Solvent solution:* acetone/bi-distilled water *Flow rate*: 0.5–1.5 mL h^−1^	287 ± 76 to 1248 ± 120 (microcapsule)	Drug delivery (NSAID: ketoprofen lysinate)	[126]
CNFs	*Solvent solution:* water *Modification*: CNFs crosslinked with PA/EP resin; microspheres crosslinked with NIPAm *Flow rate*: NR	50–150 (microsphere)	Drug delivery (anticancer drug: 5-fluorouracil)	[127]
HPC	*Solvent solution:* THF *Modification*: HPC-*g*-QCP (THF-*co*-ECH) *Flow rate*: NR	3–3.3 (microsphere)	Drug delivery (NSAID: ibuprofen)	[128]
t-CNFs	*Solvent solution:* water *Modification*: crosslinking with PA/EP resin *Flow rate*: NR	2–7 (microsphere)	Water remediation (metal ion: Cu^2+^)	[129]
t-CNFs	*Solvent solution:* water/cysteamine/FITC-dextran *Flow rate*: NR	12.1–13.8 (microsphere)	Drug delivery (model drug: FITC-dextran)	[130]

Abbreviations: AP: apple pectin; CA: cellulose acetate; CH: chitosan; CMC: carboxymethylcellulose; CNCs: cellulose nanocrystals; CNFs: cellulose nanofibrils; EP: epichlorohydrin; FITC: fluorescein isothiocyanate; HEK 293T: human embryonic kidney cells; HPC: hydroxypropyl cellulose; HPC-*g*-QCP(THF-*co*-ECH): quaternized hydroxypropyl cellulose-*g*-poly(tetrahydrofuran-*co*-epichlorohydrin) graft copolymers; MF: melamine formaldehyde; NSAID: nonsteroidal anti-inflammatory drug; NIPAm: *N*-isopropylacrylamide; NR: not reported; PA: polyamide; QA-CNFs: quaternary ammonium modified CNFs; t-CNFs: TEMPO (2,2,6,6-tetramethylpiperidinyl-1-oxyl) oxidized CNFs; THF: tetrahydrofuran; XyG: xyloglucan.

**Table 4:** Examples of spherical cellulose-based nanoparticles produced by nanoprecipitation.

Cellulosic Substrate	General Features	Diameter (nm)	Application	Ref.
CA	*Solution*: CA in acetone *Antisolvent*: water	~300 (nanosphere)	Biocide coatings (4-hexylresorcinol, triclosan)	[184]
CA	*Solution*: CA and UCNPs dispersed in a mixture of dichloromethane and acetone *Antisolvent*: water with SDS	320 ± 5 (nanocapsule)	Drug delivery (anticancer drug: DOX)	[187]
CMC	*Solution*: FA-PEG-CMC-BA/HCPT in DMSO *Antisolvent*: PBS solution (pH 7.4)	186 ± 11 (nanocapsule)	Drug delivery (anticancer drugs: BA, hydroxycamptothecine)	[159]
CMCAB	*Solution*: CMCAB/curcumin in THF *Antisolvent*: water	166.5 ± 4.2 (nanosphere)	Drug delivery (anti-inflammatory drug: curcumin)	[160]
Cotton fibres	*Solution*: cotton dissolved in NaOH/urea/thiourea (8/8/6.5 wt.%)/MB *Antisolvent*: ethanol	70–365 (nanosphere)	Drug delivery (model drug: methylene blue)	[94]
EC	*Solution*: EC/α-tocopherol or oxybenzone or avobenzone or octinoxate in ethanol *Antisolvent*: water	~50 (nanocapsule)	Cosmetics (UV-filters in sunscreens)	[161]
Kraft paper/ wastepaper cellulose	*Solution*: paper waste dissolved in NaOH/urea/thiourea (8/8/6.5 wt.%) *Antisolvent*: water	~50 (nanosphere)	Transistors and batteries	[162]
MCC	*Solution*: CE-*g*-PMMA/BA in DMSO *Antisolvent*: PBS	~120 (nanocapsule)	Drug delivery (anticancer drug: BA)	[163]
MCC	*Solution*: DAC (obtained by cellulose oxidation with sodium periodate)/oleylamine/AERhB in DMF *Antisolvent*: water	152.1 ± 0.9 156.3 ± 1.0 (nanocapsule)	Drug delivery (model drug: AERhB)	[164]
MCC	*Solution*: MCC dissolved in [C_2_mim][Ac] *Antisolvent*: acetonitrile	100–400 (nanosphere)	–	[165]
Cellulose fibres (from paper waste)	*Solution*: carboxylic CA (obtained via TEMPO oxidation and acetylation) in ultrapure water *Antisolvent*: ethanol	70–100 (nanosphere)	Drug delivery (antibiotic: penicillin G)	[166]

Abbreviations: [Bmim]Cl: 1-butyl-3-methylimidazolium chloride; [C2mim][Ac]: 1-ethyl-3-methylimidazolium acetate; AERhB: aminoethyl rhodamine; BA: betulinic acid; CA: cellulose acetate; CE-g-PMMA: cellulose-poly(methyl methacrylate) copolymer; CMC: carboxymethylcellulose; CMCAB: carboxymethylcellulose acetate butyrate; DAC: 2,3-dialdehyde cellulose; DMF: *N*,*N*-dimethylformamide; DMSO: dimethyl sulfoxide; EC: ethyl cellulose; FA-PEG-CMC-BA: folic acid-poly(ethylene glycol)-carboxymethylcellulose-betulinic acid copolymer; HCPT: hydroxycamptothecin; MB: Methylene blue; MCC: microcrystalline cellulose; PBS: phosphate buffered saline; SDS: Sodium dodecyl sulphate; TEMPO: 2,2,6,6-tetramethylpiperidinyl-1-oxyl; THF: tetrahydrofuran; UCNPs: luminescent up-conversion nanoparticles.

**Table 5:** Examples of spherical cellulose-based nanoparticles produced by emulsification processes.

Cellulosic Substrate	General Features	Diameter (nm)	Application	Ref.
CAP	*Aqueous phase*: PVA in water *Oil phase*: CAP in methyl ethyl ketone /eugenol oil/CHX (for nanocapsules) and CAP in methyl ethyl ketone/CHX (for the control nanospheres) *Emulsion type*: *o*/*w*	~248 (nanosphere) 290–324 (nanocapsule)	Drug delivery (antiseptic drug: CHX)	[167]
CMC	*Aqueous phase*: w1: PEG in water and w2: AZT/CMC in water *Oil phase*: compritol in DCM *Emulsion type*: *w*/*o*/*w*	162 ± 44 (nanocapsule)	Drug delivery (antiretroviral drug: AZT)	[168]
EC	*Aqueous phase*: PVA in water *Oil phase*: spirooxazine dye/EC in DCM *Emulsion type*: *o*/*w*	193–404 (nanocapsule)	–	[169]
EC	*Aqueous phase*: PEG/SDS in nitric acid aqueous solution *Oil phase*: EC in ethanol+benzene/linseed oil/n-decane *Emulsion type*: *o*/*w*	33–473 (nanocapsule)	Anticorrosion coatings	[141,185]
EC	*Aqueous phase*: water *Oil phase*: EC/MC in ethanol/α-mangostin *Emulsion type*: *w*/*o*	436 ± 11 (nanosphere)	Drug delivery (antiacne drug: α-mangostin)	[170]
EC	*Aqueous phase*: water/PVA or P188 or CA25 *Oil phase*: EC in ethyl acetate *Emulsion type*: *o*/*w*	165 ± 44 to 474 ± 66 (nanosphere)	Drug delivery (NSAID: piroxicam)	[171]
EC	*Aqueous phase*: PVA in water *Oil phase*: EC in ethyl acetate *Emulsion type*: *o*/*w*	147 ± 2 (nanosphere)	Drug delivery (corticosteroid drug: dexamethason)	[172]
EC	*Aqueous phase*: ethyl acetate-saturated water/TWEEN^®^ 80 *Oil phase*: EC in ethyl acetate/astaxanthin *Emulsion type*: *o*/*w*	161 ± 8 to 733 ± 7 (nanocapsule)	Delivery of bioactive compounds (carotenoid pigment: astaxanthin)	[173]

Abbreviations: AZT: zidovudine; CA25: cremophor A25; CAP: cellulose acetate phthalate; CHX: chlorhexidine; CMC: carboxymethylcellulose; DCM: dichloromethane; EC: ethyl cellulose; MC: methyl cellulose; NSAID: nonsteroidal anti-inflammatory drug; *o*/*w*: oil-in-water; P188: poloxamer 188; PEG: poly(ethylene glycol); PVA: poly(vinyl alcohol); SDS: Sodium dodecyl sulphate; TWEEN^®^ 80: polyethylene glycol sorbitan monooleate (non-ionic surfactant); w1: primary aqueous-phase; w2: secondary aqueous-phase; *w*/*o*: water-in-oil; *w*/*o*/w: water-in-oil-in-water.

**Table 6:** Examples of spherical cellulose-based nanoparticles produced by other methodologies.

Cellulosic Substrate	General Features	Diameter (nm)	Application	Ref.
MECHANICAL TREATMENTS				
Bamboo pulp	*Methodology*: high-pressure homogenization followed by oxidation and aging	~15–35 (nanocapsule)	Drug delivery (hypolipidemic drug: lovastatin)	[175]
Cellulose from pine needles	*Methodology*: cellulose oxidation with TEMPO radical and sodium periodate followed by sonication	<30 (nanosphere)	Drug delivery (anticancer drug: DOX)	[176]
Cellulose dissolving pulp (softwood) and MCC	*Methodology*: mechanical disintegration of the fibres after dissolution and regeneration of cellulose from a DES	5.6 ± 1.4 5.8 ± 1.4 (nanosphere)	Reinforcement agents	[177]
CHEMICAL AND/OR ENZIMATIC TREATMENTS				
Cotton linter	*Methodology*: acid hydrolysis followed by lipase catalysed esterification with 3-MPA	45–75 (nanosphere)	Water remediation (metal ion: Hg^2+^)	[178]
Bleached Kraft eucalyptus pulp	*Methodology*: enzymatic hydrolysis	15–40 (nanosphere)	–	[179,180]
Lyocell fibres	*Methodology*: acid hydrolysis followed by the one-pot Fischer esterification with formic acid	19–29 (nanosphere)	–	[181]
Lyocell fibres	*Methodology*: mixed acid hydrolysis (HCOOH and HCl) of the fibres followed by ultrasonic irradiation	27.0 ± 1.2 (nanosphere)	Nucleation/reinforcing agent in films for food packaging	[182]
SELF-ASSEMBLY PROCESSES				
CMC	*Methodology*: graft polymerization of CMC with DMDAAC (CMC-*g*-PDMDAAC) and encapsulation of avermectin via electrostatic interactions	~100–150 (nanocapsule)	Pesticide delivery (avermectin)	[193]
CMC	*Methodology*: graft polymerization of CMC with methyl methacrylate (CMC-*g*-PMMA), butyl acrylate (CMC-*g*-PBA) or styrene (CMC-*g*-PS), followed by emulsion to prepare avermectin/grafted polymer nanoparticles	~230 ~180 230–260 (nanocapsule)	Pesticide delivery (avermectin)	[194]
CMC	*Methodology*: graft polymerization of CMC and DMDAAC (CMC-*g*-PDMDAAC) followed by electrostatic assembly with P-Zein and encapsulation of avermectin	360 (nanocapsule)	Pesticide delivery (avermectin)	[195]
CMC	*Methodology*: shell of CMC modified with hexamethylenediamine coated on a core of Fe_3_O_4_ nanoparticle	70–120 (nanocapsule)	Drug delivery (anticancer drug: DOX)	[196]
CMC	*Methodology*: graft polymerization of CMC with ImIL (CMC-*g*-PIL) followed by coating on a core of Fe_3_O_4_ nanoparticle	39.2 ± 8.4 (nanocapsule)	Drug delivery (anticancer drug: DOX)	[197]
CMC	*Methodology*: graft polymerization of CMC with DMAEMA (CMC-*g*-PDMAEMA)	118–133 (nanocapsule)	Drug delivery (anticancer drug: paclitaxel)	[198]
HEC	*Methodology*: graft polymerization of CEHEC with adamantane (CEHEC-*g*-Ad) followed by self-assembly with GE-CD and CD-DOX	36.4 ± 2.2 (nanocapsule)	Drug delivery (anticancer drug: DOX)	[199]
HPMC	*Methodology*: graft polymerization of HPMC with PLA (HPMC-*g*-PLA)	175–216 (nanocapsule)	Drug delivery (anticancer drug: paclitaxel)	[200]
Lyocell (TENCEL™ Lyocell)	*Methodology*: carboxymethylation of TENCEL™ gel followed by homogenization in a high-pressure homogenizer	73–129 (nanosphere)	–	[203]
Lyocell (LENZING™ Lyocell)	*Methodology*: carboxymethylation of lyocell fibres followed by homogenization in a microfluidizer	16 ± 5 (TEM) 22 ± 7 (AFM) 51 ± 4 (DLS) (nanocapsule)	–	[204]
Lyocell (LENZING™ Lyocell)	*Methodology*: heterogenous modification of lyocell gel with glycidyltrimethylammonium chloride followed by mechanical shearing in a microfluidizer	30 ± 8 (AFM) 55 ± 8 (DLS) (nanocapsule)	Immunoassays (proteins: hIgG, BSA)	[205]
LAYER-BY-LAYER ASSEMBLY				
CMC	*Methodology*: assembly of 3 CMC/protamine bilayers on a silica sacrificial template, followed by surface decoration with ferrite nanoparticles	150 ± 20 (nanocapsule)	Drug delivery (anticancer drug: DOX)	[183]
CMC and QHECE	*Methodology*: LbL deposition of CMC and QHECE on a cationic vesicular template of DDAB	306 (1^st^ layer) up to 1,600 (6^th^ layer) (nanocapsules)	Potential for drug delivery	[206]

Abbreviations: Ad: adamantane; AFM: atomic force microscopy; BSA: bovine serum albumin; CD-DOX: β-cyclodextrin grafted with doxorubicin; CEHEC: carboxyethyl hydroxyethyl cellulose; CMC: carboxymethylcellulose; DDAB: dimethyldioctadecylammonium bromide; DES: deep eutectic solvent; DLS: dynamic light scattering; DMAEMA: *N*,*N*-dimethylaminoethyl methacrylate; DMDAAC: dimethyldiallylammonium chloride; DOX: doxorubicin; GE-CD: glycerol ethoxylate grafted with β-cyclodextrin; HEC: hydroxyethyl cellulose; hIgG: human immunoglobulin G; HPMC: hydroxypropyl methyl cellulose; ImIL: 1-methyl-3-(oxi-rane-2-ylmethyl)-1H-imidazol-3-ium chloride; LENZING™ Lyocell: Lenzing regenerated cellulose fibres for industrial applications; 3-MPA: 3-mercaptopropionic acid; PBA: poly(butyl acrylate); PDMAEMA: poly(*N*,*N*-dimethylaminoethyl methacrylate); PDMDAAC: poly(dimethyldiallylammonium chloride); PIL: poly(ionic liquid;) PLA: poly(lactic acid); PMMA: poly(methyl methacrylate); PS: polystyrene; P-Zein: phosphorylated zein; QHECE: quaternized hydroxyethylcellulose ethoxylate; TEM: transmission electron microscopy; TEMPO: 2,2,6,6-tetramethylpiperidinyl-1-oxyl radical; TENCEL™ Lyocell: Lenzing’s flagship brand of regenerated cellulose fibres for textiles.
